# Clinician and radiologist chest radiography interpretation variability in a high-TB-burden setting

**DOI:** 10.5588/ijtldopen.25.0544

**Published:** 2026-01-09

**Authors:** N. Navuluri, C. Kipchirchir, D. Lagat, E. Birgen, S. Kitur, N. Thielman, L.G. Que, K. Wools-Kaloustian, P.S. Kussin, T.D. Tailor

**Affiliations:** 1 Division of Pulmonary and Critical Care, Department of Medicine, Duke University School of Medicine, Durham, NC, USA;; 2 Duke Global Health Institute, Duke University, Durham, NC, USA;; 3 Moi University School of Medicine and Moi Teaching and Referral Hospital, Eldoret, Kenya;; 4 Academic Model Providing Access to Healthcare (AMPATH), Eldoret, Kenya;; 5 Division of Infectious Disease, Duke University School of Medicine, Durham, NC, USA;; 6 Division of Infectious Disease, Department of Medicine, Indiana University School of Medicine, Indianapolis, IN, USA;; 7 Division of Cardiothoracic Radiology, Department of Radiology, Duke University School of Medicine, Durham, NC, USA.

**Keywords:** tuberculosis, chest radiograph, Kenya, CXR, lung disease, radiology

Dear Editor,

Chest radiography (CXR) is a quick, accessible, and low-cost imaging test used in the assessment and monitoring of many acute and chronic lung diseases, including pneumonia, lung cancer, obstructive lung disease, pulmonary oedema, interstitial lung disease, and TB. The utility of CXR relies on accurate interpretation, which may be affected by technical and human factors. Interpretability depends on projection of X-rays through multiple organs, absence of superimposed findings, and high-quality equipment. Reader experience, training, and environmental factors influencing fatigue, concentration, and interruptions may also affect interpretation.^1,2^ Inter-reader agreement, or the extent of concordance among multiple readers interpreting the same CXR, is important for both clinical and research decision making. Agreement impacts the reliability and validity of findings, which have downstream impacts on clinical diagnoses and treatment, as well as research reproducibility and significance. For example, CXRs are used to screen for active TB in clinical and community settings and to determine TB severity scores in research studies.^3,4^ CXRs are also recommended for assessment of lung sequelae following completion of TB treatment and as a diagnostic test for post-TB lung disease.^5,6^ However, CXR interpretation across radiologists can be highly variable, with studies showing fair to moderate agreement on most findings.^7,8^ Furthermore, many resource-limited settings have a limited number of radiologists, with estimates ranging from 0.2 to 5.0 radiologists per 1 million people in sub-Saharan Africa, compared to 68 per million in Japan and over 100 per million in the United States (US).^9^ Thus, in low-resourced settings, clinicians often serve as front-line interpreters of CXRs, yet inter-reader agreement between clinicians and radiologists has not been widely assessed for adult CXRs.

Understanding inter-reader agreement of CXR interpretation between clinicians and radiologists for chronic lung disease can help determine reliability of findings clinicians use to guide treatment and best methods for analysing CXR data. Our purpose was to determine inter-reader agreement between pulmonary and critical care clinicians and thoracic radiologists in the US and Kenya.

The study cohort has been previously described in detail, but briefly were adults (≥18 years) admitted to the inpatient medical wards at Moi Teaching and Referral Hospital in Eldoret, Kenya, where there are approximately 36,000 CXRs performed annually.^10^ Using a procedure adapted from other studies utilising remote radiographical interpretation in resource-limited settings, participants’ de-identified CXRs were placed on a standard lighted viewing box and photographed using a study electronic tablet equipped with an 8.0-megapixel camera.^11,12^ Images were uploaded to a secure cloud storage system hosted at Duke University. An adapted standard reporting form was developed based off the chest radiograph reading and recording system and tailored for non-TB-specific findings.^13^

Two US-trained pulmonary and critical care physicians (1 senior fellow with 7 years of CXR reading experience [NN] and 1 senior attending with 35 years of CXR reading experience [PSK]) read CXRs together simultaneously and provided a single read which was intentionally done to mirror clinical practice in academic settings. One US-trained radiologist (TDT) with subspecialty training in cardiothoracic radiology and 15 years of CXR interpretation experience and one Kenyan trained radiologist (CK) with subspecialty training in thoracic interventional radiology and 14 years of interpretation experience also read CXRs. These three interpretations were rendered independently, and readers were blinded to the other two interpretations as well as to participants’ clinical data and diagnoses. Interpretations were collected and managed using REDCap electronic data capture tools hosted at Duke University. Training regarding the reporting form was provided to all readers prior to the actual reading.

Fleiss Kappa statistics were used to investigate inter-rater agreement between the three reads for each interpretation variable.^14^ The following guidelines were used for interpretation of kappa coefficients: <0, poor agreement; 0–0.20, slight; 0.21–0.40, fair; 0.41–0.60, moderate; 0.61–0.80, good; and 0.81–1.00, excellent. Kappa values with 95% confidence intervals and *z* test derived *P* values are presented. SPSS version 29.0.2.0 was used for all analyses. All study procedures were approved by the ethical review boards at Duke University (Pro00100397) and Moi University (IREC/2019/72).

Among 243 available CXRs, 240 (98.8%) were interpretable by all readers. The three uninterpretable CXRs included one underpenetrated image, one severely rotated image, and one with overlying structure obscuring thoracic anatomy. Table shows the percentage agreement and kappa statistics with 95% confidence intervals for each interpretative variable. There was good agreement on presence or absence of parenchymal abnormalities and pneumothorax. There was moderate agreement on overall findings of abnormal versus normal, central abnormalities, infiltrates, infiltrate side, infiltrate position, infiltrate extent, presence or absence of nodules, nodule size, pleural effusion, tracheal deviation, mediastinal shift, and cardiac silhouette enlargement. There was fair agreement on hyperinflation, cavitary lesions, fibrosis, and adenopathy calcification type. There was slight agreement on presence or absence of adenopathy, adenopathy location, and pulmonary artery enlargement.

**Table.** Inter-reader agreement between radiologists and clinicians on chest radiograph (CXR) findings.

Variable^**A**^	% agreement	Kappa	Lower 95% CI	Upper 95% CI
Overall findings (normal vs. abnormal)	87.92%	0.592	0.519	0.665
Parenchymal abnormalities (yes vs. no)	85.83%	0.695	0.622	0.768
Central abnormalities (yes vs. no)	71.25%	0.559	0.486	0.632
Parenchymal abnormalities				
Cavitary lesions (yes single, yes multiple, no, possible)	82.08%	0.308	0.255	0.360
Infiltrates (yes vs. no)	67.92%	0.509	0.436	0.582
Infiltrate type: consolidative, interstitial, or both	26.25%	0.253	0.209	0.296
Infiltrate side: left, right, or both	46.67%	0.418	0.369	0.466
Infiltrate position: apical, basal, or both	45.83%	0.436	0.388	0.484
Infiltrate extent: focal or diffuse (≥50% of hemithorax)	44.58%	0.408	0.355	0.460
Nodules (yes single, yes multiple, no, possible)	70.42%	0.419	0.367	0.471
Nodule size: <1, 1–5, >5 cm	72.92%	0.443	0.383	0.504
Fibrosis (yes, no, possible)	64.17%	0.334	0.278	0.390
Pleural effusion (yes, no, possible)	68.33%	0.600	0.536	0.665
Pneumothorax (yes, no, possible)	94.17%	0.681	0.619	0.742
Central structure abnormalities				
Adenopathy (yes, no, possible)	59.58%	0.163	0.106	0.220
Adenopathy location (hilar, mediastinal, both)	59.58%	0.195	0.145	0.245
Adenopathy type (calcified, not calcified, both)	61.67%	0.234	0.168	0.300
Tracheal deviation (yes, no)	83.33%	0.597	0.524	0.670
Mediastinal shift (yes, no)	90.00%	0.564	0.491	0.637
Cardiac silhouette enlargement (yes, no, possible)	67.92%	0.585	0.523	0.647
Pulmonary artery enlargement (yes, no, possible)	51.25%	0.183	0.128	0.238
Hyperinflation (yes vs. no)	71.25%	0.371	0.298	0.444

Red = poor or slight agreement; orange/amber = fair or moderate agreement; green = good or excellent agreement.

CI = confidence interval.

A Each variable represents a distinct question on the CXR interpretation form, and response options are shown in parentheses.

These findings illustrate the high variability in inter-reader agreement for CXR interpretation and highlight the types of findings which may be more reliable across interpreters. For example, the good agreement on the presence or absence of parenchymal abnormalities and pneumothorax and moderate agreement on overall findings, central abnormalities, and infiltrates suggest that these more general findings may be among the most reliable when using CXRs to rule out lung disease or for end-of-TB-treatment assessment. The level of agreement also suggests that clinician interpretations of these findings may be considered acceptable in settings where radiologist availability is limited. In contrast, the fair agreement on cavitary lesions and slight agreement on adenopathy and pulmonary artery enlargement suggests that a single interpreter read of these, whether by a radiologist or a clinician, may not be reliable for clinical or research decision making and raises potential concerns about using these findings to grade disease severity or for risk-stratification. The levels of agreement in this study are consistent with other studies which show that agreement on CXR interpretation is generally fair to moderate, with better agreement for findings such as alveolar infiltrates or cavitary lesions, but fair or poor agreement for findings such as air bronchograms and hilar adenopathy and infiltrate type.^7,8,15^

Study strengths include the large sample size and the inclusion of clinicians and radiologists trained in different settings. Limitations include that the original CXRs were not available; rather, readers interpreted photographed CXRs, which could affect interpretation integrity. The multiple response options provided for interpreting cavitary lesions and nodules may have negatively affected agreement. We also were unable to compare CXR findings to CT findings or include computer-aided detection interpretations in this study. Given that computer-aided detection models are developed from human interpretations, the degree of variation in inter-reader agreement may affect the accuracy and reliability of those models.^16^

In conclusion, our findings provide a road map for future lung disease research in high-TB-burden, low-resource settings. Agreement of CXR interpretations between radiologists and clinicians is greatest for broader findings and lower for more detailed findings, which highlights the limitations of a single CXR reader for more specific chronic disease findings.
